# Prospective Follow-Up of Adolescents With and at Risk for Depression: Protocol and Methods of the Identifying Depression Early in Adolescence Risk Stratified Cohort Longitudinal Assessments

**DOI:** 10.1016/j.jaacop.2023.11.002

**Published:** 2023-12-14

**Authors:** Jader Piccin, Anna Viduani, Claudia Buchweitz, Rivka B. Pereira, Aline Zimerman, Guilherme R. Amando, Victor Cosenza, Leonardo Z. Ferreira, Natália A.G. McMahon, Ramásio F. Melo, Danyella Richter, Frederico D.S. Reckziegel, Fernanda Rohrsetzer, Laila Souza, André C. Tonon, Marina Tuerlinckx Costa-Valle, Zuzanna Zajkowska, Ricardo Matsumura Araújo, Tobias U. Hauser, Alastair van Heerden, Maria Paz Hidalgo, Brandon A. Kohrt, Valeria Mondelli, Johnna R. Swartz, Helen L. Fisher, Christian Kieling

**Affiliations:** a Universidade Federal do Rio Grande do Sul (UFRGS), Porto Alegre, Brazil; b Prodia - Child & Adolescent Depression Program, Hospital de Clínicas de Porto Alegre (HCPA), Porto Alegre, Brazil; c Laboratório de Cronobiologia e Sono, Hospital de Clínicas de Porto Alegre, Porto Alegre, Brazil; d Universidade Federal de Pelotas (UFPEL), Pelotas, Brazil; e King’s College London, Institute of Psychiatry, Psychology & Neuroscience, London, United Kingdom; f National Institute for Health and Care Research Maudsley Mental Health Biomedical Research Centre, South London and Maudsley NHS Foundation Trust, London, United Kingdom; g ESRC Centre for Society and Mental Health, King’s College London, London, United Kingdom; h Max Planck University College London Centre for Computational Psychiatry and Ageing Research, University College London, London, United Kingdom, Wellcome Centre for Human Neuroimaging, University College London, London, United Kingdom and with Eberhard Karls University of Tübingen, Tübingen, Germany; i Human and Social Development, Human Sciences Research Council, Pietermaritzburg, South Africa and Medical Research Council/Wits Developmental Pathways for Health Research Unit, University of the Witwatersrand, Johannesburg, South Africa; j The George Washington University, Washington, DC, United States; k University of California, Davis, Davis, CA, United States

**Keywords:** depression, adolescence, risk score, cohort, digital phenotyping

## Abstract

**Objective:**

To present the protocol and methods for the prospective longitudinal assessments—including clinical and digital phenotyping approaches—of the Identifying Depression Early in Adolescence Risk Stratified Cohort (IDEA-RiSCo) study, which comprises Brazilian adolescents stratified at baseline by risk of developing depression or presence of depression.

**Method:**

Of 7,720 screened adolescents aged 14 to 16 years, we recruited 150 participants (75 boys, 75 girls) based on a composite risk score: 50 with low risk for developing depression (LR), 50 with high risk for developing depression (HR), and 50 with an active untreated major depressive episode (MDD). Three annual follow-up assessments were conducted, involving clinical measures (parent- and adolescent-reported questionnaires and psychiatrist assessments), active and passive data sensing via smartphones, and neurobiological measures (neuroimaging and biological material samples). Retention rates were 96% (Wave 1), 94% (Wave 2), and 88% (Wave 3), with no significant differences by sex or group (*p* > .05). Participants highlighted their familiarity with the research team and assessment process as a motivator for sustained engagement.

**Discussion:**

This protocol relied on novel aspects, such as the use of a WhatsApp bot, which is particularly pertinent for low- to-middle-income countries, and the collection of information from diverse sources in a longitudinal design, encompassing clinical data, self-reports, parental reports, Global Positioning System (GPS) data, and ecological momentary assessments. The study engaged adolescents over an extensive period and demonstrated the feasibility of conducting a prospective follow-up study with a risk-enriched cohort of adolescents in a middle-income country, integrating mobile technology with traditional methodologies to enhance longitudinal data collection.

## Plain language summary

This article details the study protocol and methods used in the longitudinal assessment of 150 Brazilian teenagers with depression and at risk for depression as part of the Identifying Depression Early in Adolescence Risk Stratified Cohort (IDEA-RiSCo). Over 3 years, the authors collected clinical and digital data using innovative mobile technology, including a WhatsApp bot. Most adolescents participated in all the study phases, showing feasibility of prospective follow-up in a middle-income country. This approach allowed for a deeper understanding of depression in young populations, particularly in areas where mental health research is scarce.

Depressive disorders constitute a leading cause of disability among youth across the globe.^1^ To effectively reduce the burden associated with depression throughout the lifespan, efforts beyond treatment are needed, with prevention initiatives representing a compelling strategy, especially when targeting younger individuals.^2^ However, our ability to design and to implement targeted preventive measures has been hindered by the difficulties of identifying increased risk for depression in youth. Moreover, there is still limited knowledge in terms of how the disorder develops in adolescence, and of the aspects that distinguish individuals who are at the highest or lowest risk for depression.^3^ In that sense, previous research suggests that using predictive models with multivariate scores to stratify individual-level risk constitutes a key strategy.^4^^,^^5^ Although an increasing number of models to predict individualized risk of mental health outcomes is available, most prognostic studies use databases *post hoc* that were not specifically designed to test the models.^5^^,^^6^ In addition, there are few prospectively designed studies using risk stratification as inclusion criteria to address trajectories of different risk groups for depression.

Two important characteristics of depression—an (often) episodic nature, with remittance and recurrence of depressive episodes across the life course,^7^^,^^8^ and a heterogeneous presentation,^9^ with individual variations in terms of which and how different signs and symptoms manifest—make the longitudinal approach an essential aspect for consideration by researchers in the field. Furthermore, depressive disorders frequently emerge in adolescence, in a period of intense psychosocial change^10^ and neurobiological maturation,^11^ with important differences in relation to adult depression, particularly in terms of clinical trajectories, treatment response, and outcomes across the life course.^12^^,^^13^

To expand the understanding regarding the phenotypic and neurobiological profiles associated with increased risk or presence of depression in adolescence, we have previously performed the Identifying Depression Early in Adolescence Risk Stratified Cohort (IDEA-RiSCo) study, an in depth study of multiple neurobiological, psychological, and environmental measures associated with the risk of developing, and with the presence of, depression in adolescence, with a focus on immune/inflammatory and neuroimaging markers.^14^ In that initial work, 7,720 adolescents aged 14 to 16 years were screened in 101 public schools in Porto Alegre, Brazil, using an empirically developed sociodemographic composite risk score.^14^ From this sample, 150 adolescents were recruited who were also clinically assessed for current and lifetime depressive disorders.^14^ Participants were stratified into 3 groups: 50 with low risk for developing depression (LR), 50 with high risk for developing depression (HR), and 50 with an active untreated major depressive episode (MDD). Detailed clinical characteristics of the IDEA-RiSCo sample (IDEA-RiSCo cohort) at baseline have been described elsewhere.^14^

Risk stratification was operationalized using the Identifying Depression Early in Adolescence Risk Score (IDEA-RS).^15^ The IDEA-RS is a multivariable model developed by our group to estimate individual risk in early adolescence of developing depression at age 18 years. It was designed to be easily obtained directly from the adolescent in a brief assessment comprising 11 sociodemographic predictor variables: biological sex, skin color, drug use, school failure, social isolation, fight involvement, poor relationship with father, mother, and between parents, childhood maltreatment, and having run away from home. IDEA-RS exhibited a good discriminative performance to stratify adolescents at age 15 years in terms of individual risk for developing depression at age 18 years, as measured by a C-statistic of 0.78 (95% CI: 0.73-0.82) in the development sample.^15^ IDEA-RS was subsequently evaluated across several sociocultural and economic contexts including the United Kingdom, Nigeria, New Zealand, Nepal, and North America, reaching beyond chance discriminative ability in all settings (C-statistics between 0.59 and 0.73).^15^, ^16^, ^17^, ^18^

Following confirmation of this informative discriminative performance to estimate the individualized risk for depression across 5 continents, a prospective analysis of the performance of the IDEA-RS to assess the development of depression was planned for the IDEA-RiSCo cohort, a phenotypically refined sample, stratified by risk of developing depression.^14^ Prospective, longitudinal assessments of the IDEA-RiSCo sample may provide insights into the trajectories of different risk groups during a crucial developmental period; evaluating the progression of adolescents in the LR and HR groups is likely to provide essential information to clarify the differences between individuals who do and do not convert to depression.

Furthermore, although 90% of the world’s adolescents live in low- and middle-income countries (LMICs), most research on adolescent depression is still conducted in high-income countries (HICs), leaving a significant gap in knowledge.^19^ In that sense, longitudinal studies represent important resources to capture the overall trajectories of adolescents stratified by risk of developing depression in LMICs, providing a means for generalizations, group comparisons and the monitoring of changes within a macro developmental dimension.^20^ Also, most existing longitudinal datasets encompassing mood disorders in adolescence seem unable to capture granular changes or fluctuations in symptom patterns that occur over short periods of time as individuals go about their lives. Thus, leveraging the capacity of digital devices, such as smartphones and wearables, to gather repeated information in a person's natural environment while minimizing recall bias may constitute an important avenue of inquiry. The analysis of active and passive data from behavioral information obtained from mobile devices allows an ecologically valid identification of context-based digital phenotypes,^21^ and novel statistical methods are capable of accounting for both individual-level differences and the behavior of a group over time.^22^^,^^23^ At a global level, 1 in 3 Internet users is under 18 years of age, and smartphones are the most popular devices used to go online.^24^ This, combined with the advances in technology and integration of better, more accurate sensors in mobile phones, provides an unparalleled potential for conducting research in real-world settings in a more practical, accessible, and acceptable way. Moreover, using smartphones to collect data may also address gaps between HICs and LMICs: it enables cost-effective data collection that has the potential to improve early identification of mental health conditions.^25^ Hence, to shed light on the characteristics and course of the risk and presence of depression throughout adolescence, a combination of traditional phenotypic assessment with digital phenotyping seems especially useful.^26^

Considering these aspects, we here present a detailed account of the protocol and methods designed for prospective mood assessment across time among Brazilian adolescents included in the IDEA-RiSCo cohort. In addition to the traditional phenotypic assessment focusing on clinical evaluations, the follow-up assessments also leveraged the use of innovative mobile technologies to collect intensive longitudinal information in a digital phenotyping approach.^26^ Procedures for clinical and phenotypic assessments are described for 4 waves of data collection, as well as for peripheral biological sampling and neuroimaging assessments. We also present initial results in terms of feasibility and retention rates for each procedure and collection point.

## Method

### Ethics Approval

This study was approved by the Research Ethics Committee of Hospital de Clínicas de Porto Alegre and by the Brazilian National Ethics in Research Commission (CAAEs 17574719.0.0000.5327, 41801121.0.0000.5327, and 50473015.9.0000.5327). Supplemental approval was granted by the Institutional Review Board of University of California at Davis (1218177-4) and the Psychiatry, Nursing and Midwifery Research Ethics Subcommittee of King’s College London (LRS-17/18-8327).

All adolescents and caregivers provided written consent in the baseline assessment and in each wave of follow-up. For adolescents under the age of 18 years, legal guardians were required to provide formal consent. If the participant reached age 18, collateral information from a parent (or another relevant adult) was collected only with the consent of the participant. In situations in which participants’ scores and/or responses indicated risk, they were referred to emergency services or specialized care according to Brazilian legislation. Participants received no financial incentive for taking part in the study but were compensated for expenses related to their participation (eg, transportation to data collection site, mobile data use). Also, smartphones were lent to participants who did not own a smartphone during the study period.

### Study Design and Participants

The IDEA-RiSCo sample included 150 adolescents (75 boys, 75 girls) aged 14 to 16 years (mean = 15.6 [SD = 0.82]) at baseline (Wave 0-W0). Clinical data were collected at 3 additional waves during the follow-up period lasting 3 years, to mimic the original age interval used in the development of the IDEA-RS. Recruitment for Wave 1 (W1), Wave 2 (W2), and Wave 3 (W3) occurred 12 months, 24 months, and 32 months after baseline (W0), respectively. At the last follow-up, participants were aged 17 to 19 years (mean = 18.0 [SD = 0.80])

**Figure 1:**
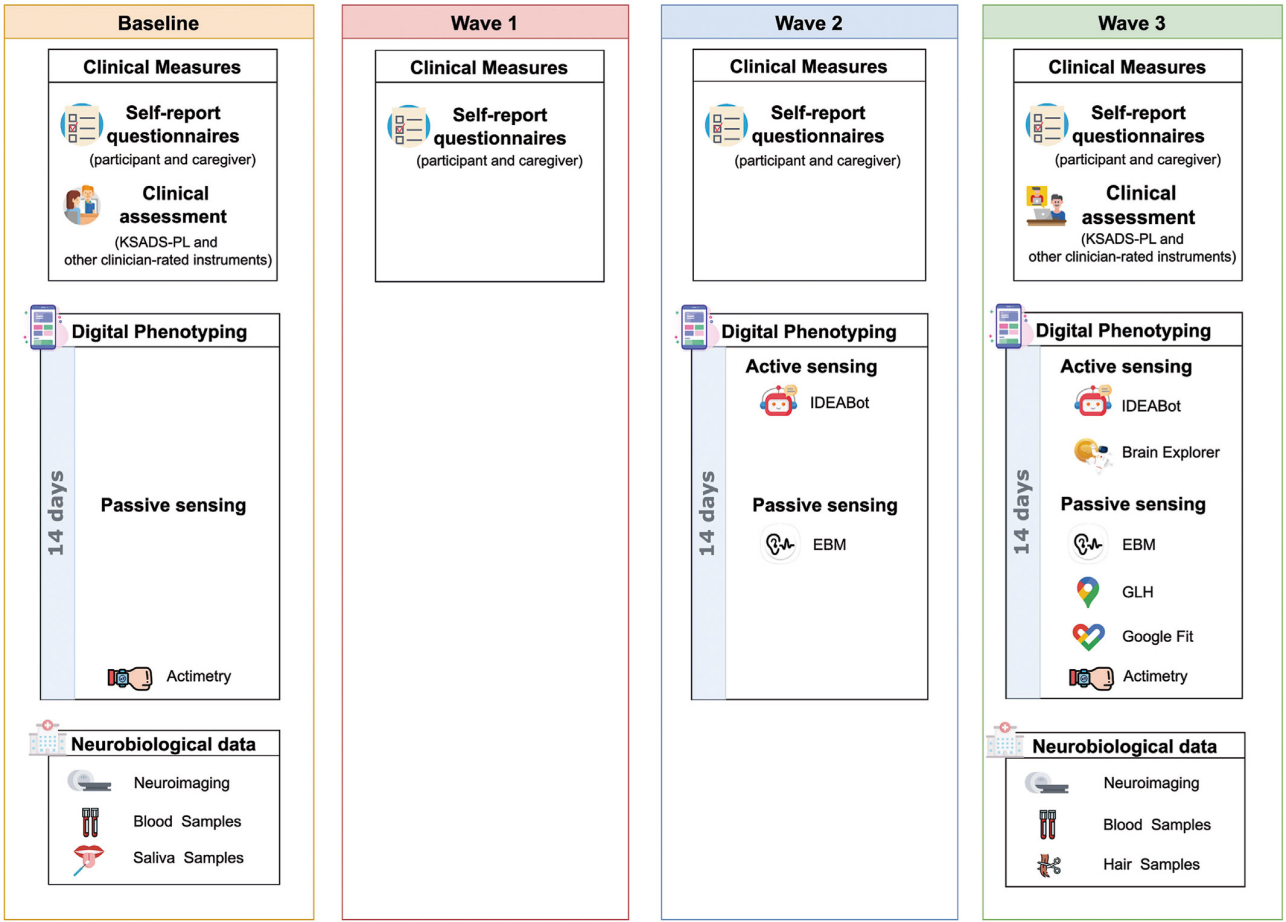
Timeline of Each Data Collection Mode Across the 3 Waves ***Note:****EBM = electronic behavioral monitoring; GLH = Google Location History; IDEABot = The Identifying Depression Early in Adolescence Chatbot; K-SADS-PL = Schedule for Affective Disorders and Schizophrenia for School-Age Children—Present and Lifetime version.*

IDEA-RiSCo participants were stratified for risk using the IDEA-RS to estimate the probability of developing depression in 3 years. Because the incidence of depression is higher in girls in comparison to boys, sex-specific IDEA-RS models were generated to avoid over-representation of female participants in the HR group and of male participants in the LR group. At baseline, participants were interviewed by a board-certified child and adolescent psychiatrist using the Schedule for Affective Disorders and Schizophrenia for School-Age Children—Present and Lifetime Version (K-SADS-PL)^27^ and confirmed for inclusion into 1 of 3 groups: 50 low-risk participants with no current or lifetime history of depression who were at or below the 20th percentile on the IDEA-RS (LR); 50 high-risk participants with no current or lifetime history of depression who were at or above the 90th percentile on the IDEA-RS (HR); and 50 participants with current and untreated depression who were at or above the 90th percentile on the risk score (MDD). At the baseline assessment, further phenotypic, peripheral biological samples, and neuroimaging assessments were performed as described in Figure 1 and in Kieling *et al.* (2021).^14^

### Follow-Up Waves and Data Collection Procedures

The 3 follow-up waves were designed for stepwise increase in the intensity of digital and mobile data collection, as described below. A timeline with details of each data collection mode across the 3 waves is presented in Figure 1. For W1 and W2, participants were recruited if they responded to the invitation within a 2-month window in relation to their expected collection date for each wave (11-13 months and 23-25 months after baseline for W1 and W2, respectively). This was done to ensure that all were followed-up at comparable intervals. Given that Wave 3 was the final data collection point, and to improve participant retention, a larger response window of 6 months after the expected data collection date (30-36 months after baseline) was considered acceptable.

#### Wave 1 (W1)

We aimed to reassess depressive symptomatology approximately 1 year after the baseline assessment. All participants and their caregivers were recruited to electronically complete self-report online questionnaires (Table 1). Participants were contacted by phone and/or text messages, and a Web link to the questionnaire was sent separately to adolescents and caregivers.

**Table 1:** Clinical Instruments Administered in Each Wave of the Identifying Depression Early in Adolescence Risk Stratified Cohort (IDEA-RiSCo) Study

	W0	W1	W2	W3
**Adolescent self-report**				
Mood and Feelings Questionnaire—Child (MFQ-C)	✅	✅	✅	✅
*DSM-5* Self-rated Level 1 Cross-cutting Symptom Measure (CCSM-C)	✅	✅	✅	✅
Patient Health Questionnaire—Adolescent (PHQ-A)	✅	✅	✅	✅
Brazilian Criterion of Economic Classification (ABEP)	✅			✅
Coronavirus Health and Impact Survey (CRISIS)				✅
The Alcohol, Smoking and Substance Involvement Screening Test (ASSIST)				✅
Parental Bonding Instrument (PBI)	✅			✅
Childhood Trauma Questionnaire - Child (CTQ-C)	✅			✅
Snaith-Hamilton Pleasure Scale (SHAPS)	✅			✅
Affective Reactivity Index—Child (ARI-C)	✅			✅
Spence Children's Anxiety Scale—Child (SCAS-C)	✅			✅
Youth Strength Inventory—Adolescent Self-Report (YSI-A)	✅			✅
Adapted Resilience Scale (ARS)	✅			✅
Borderline Personality Features Scale for Children (BPFSC)				✅
The Munich ChronoType Questionnaire (MCTQ)	✅			✅
Puberty and Phase Preference Scale (PPPS)	✅			✅
Sleep Hygiene Index (SHI)	✅			✅
Athens Insomnia Scale (AIS)	✅			✅
**Adolescent clinician-administered**				
Schedule for Affective Disorders and Schizophrenia for School-Age Children—Present and Lifetime version (K-SADS-PL)	✅			✅
Child Depression Rating Scale—Revised (CDRS-R)	✅			✅
Clinical Global Impression—Severity Scale (CGI-S)	✅			✅
Children's Global Assessment Scale (CGAS)	✅			✅
Columbia Suicide Severity Rating Scale (C-SSRS)	✅			✅
Insomnia Severity Index (ISI)	✅			✅
**Caregiver-report**				
Mood and Feelings Questionnaire—Parent (MFQ-P)	✅	✅	✅	✅
*DSM-5* Self-rated Level 1 Cross-cutting Symptom Measure—Parent (CCSM-P)	✅	✅	✅	✅
Affective Reactivity Index—Parent (ARI-P)	✅			✅
Youth Strength Inventory—Parent (YSI-P)	✅			✅
Spence Children's Anxiety Scale—Parent (SCAS-P)	✅			✅
Mood and Feelings Questionnaire—Parent Self-report (MFQ-A)	✅			✅

#### Wave 2 (W2)

The aim of the second wave was to evaluate depressive symptomatology status and to collect remote data over a 14-day period. Participants and caregivers again completed self-report online questionnaires (Table 1). Moreover, a digital phenotyping component was added, including passive data sensing and active data collected via a chatbot. Passive data also included Global Positioning System (GPS), accelerometer/gyroscope and ambient audio data collected using a smartphone app, and the chatbot used for active collection of intensive longitudinal data on mood was operationalized via WhatsApp. Both passive and active data collection occurred simultaneously throughout the same 2-week period.

#### Wave 3 (W3)

At the final wave, we aimed to perform a detailed phenotypic assessment and a new 14-day remote data collection period. Participants and caregivers were again invited to complete a set of online self-report questionnaires that consisted of measurements about depressive symptoms and other mental health domains (Table 1). Following completion of the online questionnaires, participants were invited to take part in a face-to-face assessment, including a blinded diagnostic interview, collection of peripheral biological samples, and neuroimaging acquisition. As in W2, participants also took part in a smartphone-based active and passive data sensing period, including assessments through the WhatsApp chatbot, as well as GPS, accelerometer/gyroscope, and ambient audio data collection, similarly to W2. In addition, cognitive data were collected from gamified tasks during the remote data collection period through the Brain Explorer application. Information on sleep and biological rhythms was also collected using self-reported and actimetry data.

Following the model proposed by Wisniewski *et al.* (2020),^28^ the present protocol study included research team members who offered non-clinical support around the apps used by participants throughout the research protocol. They were able to troubleshoot simple technical app issues while guaranteeing personal, effective communication with participants. The assignment of a Digital Navigator (DN) for each participant enabled close monitoring and quick response to problems and/or concerns that might have arisen during the participation, thereby avoiding non-compliance.

Adolescents were invited to take part in all follow-up collection waves, regardless of whether they had participated in previous ones. To improve retention, the DNs actively contacted participants and their caregivers via phone calls, text messages, e-mail, and/or social media. In each wave, DNs made at least 5 attempts to contact participants/caregivers (at various times of day, on various days) for 2 weeks. Another DN would perform the same contact procedure for another 2 weeks if previous attempts were unsuccessful. If no contact was established, the accuracy of contact information for participants was checked with their school. If contact was not possible after the contact information check, the available home address was visited.

DNs were trained with core smartphone skills and basic troubleshooting for the applications used in this study. They were also able to contact a technical support team for consultations. Because they were research team members, they were aware of the information necessary to explain to participants about the types of data being collected, as well as the study’s objectives. Participants were in direct contact with DNs, notifying them of problems that might occur.

To manage participants' progress during the data collection period for W2 and W3, a Web-based dashboard was used. The DNs received training on administrator dashboard guidelines and operations to monitor the participants throughout the process. This monitoring included basic information on the completion/non-completion of digital interactions and the number of overall signals from apps. These researchers, however, intervened with data collection only when participants’ signals on apps were extremely low or nonexistent for more than 48 hours. In these cases, DNs contacted participants to troubleshoot errors in the functionality of the applications and/or smartphones. Only 1 intervention during the data collection process was made, even if the problem persisted afterward. Effective monitoring and support were established in weekly staff meetings to manage any issues that could occur during the study.

### Measures

#### Clinical Assessment

During W0 and W3, participants underwent clinical assessment of mood disorders and comorbid diagnoses using the K-SADS-PL.^27^ For both data collection points, interviews were performed by board-certified psychiatrists who received prior inter-rater reliability training on the instrument and were blind to the participant’s LR/HR/MDD group status (to avoid any bias during W3 interviews, different psychiatrists performed W0 and W3 assessments). For both waves, the best estimate of diagnoses for each participant was reviewed by an experienced child and adolescent psychiatrist to confirm and ensure diagnostic consistency. Whereas the W0 diagnostic interviews were conducted in person with both the participant and their primary caregiver, at W3 all diagnostic interviews were performed online via telemedicine with information obtained exclusively from the participants. To standardize the clinical assessment process, all participants were invited to conduct the psychiatric interview online at Hospital de Clínicas de Porto Alegre (HCPA) in a private room equipped with headphones and a computer to ensure consistency and privacy. For participants who were unable to come to the hospital, the option of conducting the evaluation online from home was offered to them.

Further self- and clinician-administered instruments used with the participants and their caregivers at each collection wave are summarized in Table 1.

#### Digital Phenotyping

During W2 and W3, we conducted active and passive data sensing for digital phenotype assessment over a 14-day period. Smartphones, the most widely adopted method of digital data collection, have been used in different populations, and have had their feasibility assessed among individuals with MDD.^29^ In addition, among 9- to 17-year-old Brazilians, 93% are regular Internet users, and 93% of these use mobile phones to access the Internet.^30^

In our study, the participants’ own smartphones were used for remote data collection whenever possible. To explain the installation of and the features of apps used for digital data collection and digital phenotyping, animated videos were produced for participants (Supplements 1, 2, and 3, available online).

**Figure 2:**
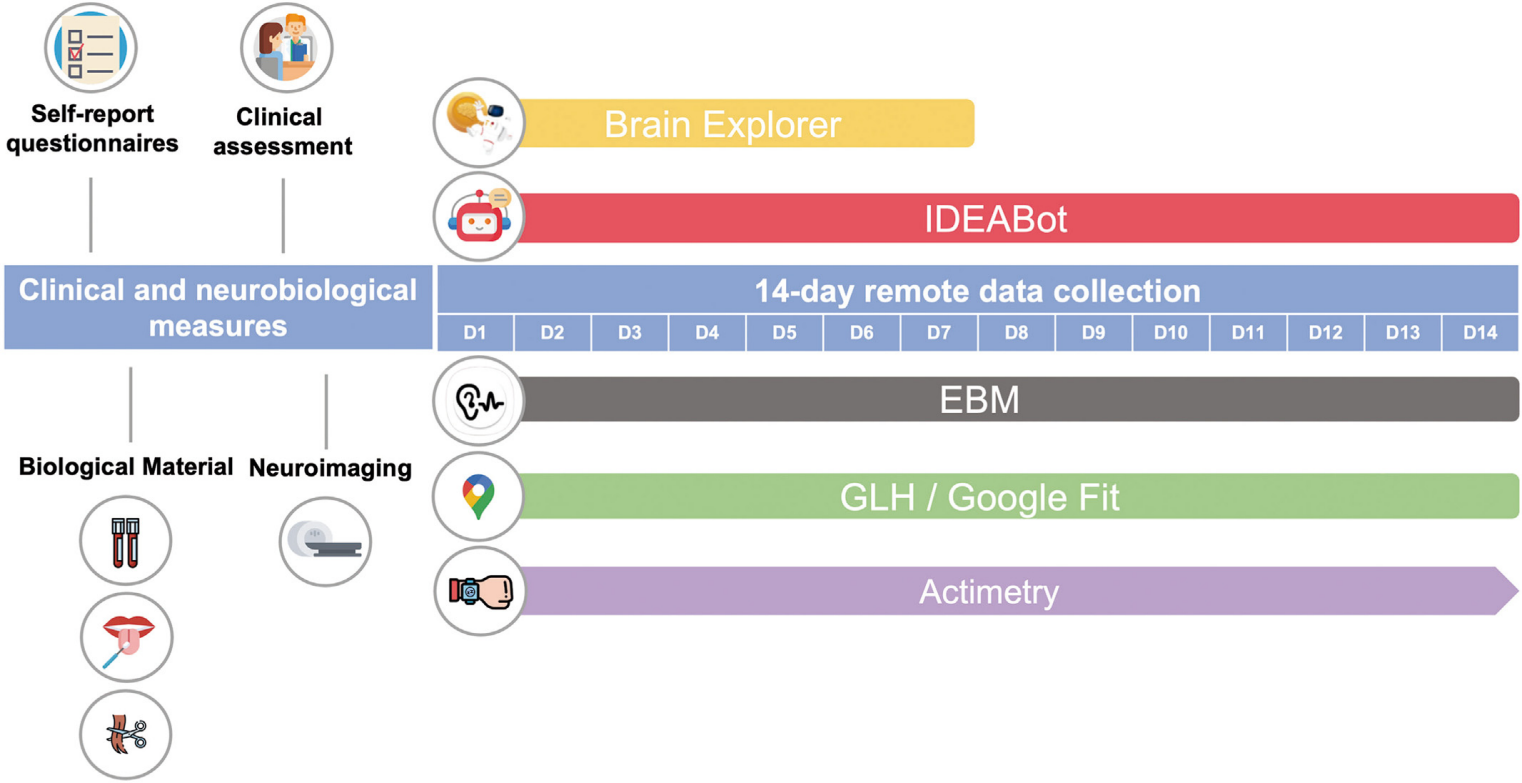
Timeline of Active and Passive Digital Phenotyping Acquisition Procedure ***Note:****Self-report questionnaires were completed by participants and caregivers at all data collection waves; clinical assessments were performed in person at W0 and via telemedicine at W3 and included K-SADS-PL and other clinician-administered instruments; biological material collection included blood samples at W0 and W3, saliva samples at W0, and hair samples at W3; neuroimaging was performed at W0 and W3; IDEABot was used to collect active sensing data at W2 and W3; the EBM app was used to collect passive sensing data at W2 and W3; GLH/Google Fit were used to collect passive sensing data at W3; actimetry was used to collect rest–activity data linked to the add-on study CHRONO-IDEA during 14 days at W0 and 23 days at W3; the Brain Explorer app was used to collect remote cognitive data during W3. EBM = Electronic Behavioral Monitoring; GLH = Google Location History; IDEABot = The Identifying Depression Early in Adolescence Chatbot; K-SADS-PL = Schedule for Affective Disorders and Schizophrenia for School-Age Children—Present and Lifetime version; W0 = Wave 0; W1 = Wave 1; W2 = Wave 2; W3 = Wave 3.*

A summary of data collection procedures across the 3 waves is provided in Figure 1. Additional information about the digital phenotyping procedures is shown in Figure 2.

#### Active Sensing Data

IDEABot is a WhatsApp-based conversational agent designed by our team to collect active momentary assessments from adolescents through text and audio messages. In the IDEA-RiSCo study, the IDEABot was programmed to collect data over a 14-day period, coinciding with the time frame during which other active and passive detection applications were also collecting data.^31^ It leverages an already-existing application—WhatsApp—and its default features to capture both real-time information on mood and to prompt audio responses from participants. The IDEABot was devised with the aim of minimizing issues regarding participant burden and to sustain engagement and retention through the use of an existing app that was already present in the daily lives of the vast majority of our target population: a recent study by our group shows that 81% of adolescents in Porto Alegre report using WhatsApp at least once every hour.^32^

The IDEABot thus constitutes a frugal innovation tool that takes advantage of human-like conversation features to assess psychological constructs in a scalable, systematic fashion.^33^ Moreover, the chatbot not only collects textual data from self-reported ratings and scales, but also prompts and collects audio recordings, from which both textual (through transcription) and acoustic features can be extracted. The IDEABot performs pre-scripted interactions that follow a time-contingent sampling and require audio or text responses from participants, deciding how to respond back based on exact text matches or recording duration. The content of recordings is not analyzed by the chatbot and is stored on a secure server for subsequent use.

An initial generic message (eg, hello) sent by participants was needed to activate the chatbot (because of WhatsApp’s technical requirements). To explain the bot’s functioning as well as its features, an animated video was sent to the participants.^31^ In addition, functioning was reviewed at the first interaction with the IDEABot. This step is critical for both standardization of instructions given to users and ensuring that participants were aware of the nature of the conversation, avoiding misconceptions (such as beliefs that the bot is a person or that the audios will be heard immediately). For more details on the development and functioning of the chatbot, see Viduani *et al.* (2023).^31^

#### Remote Cognitive Assessment

To collect remote cognitive data during W3, we selected the Brain Explorer application, developed by the Developmental Computational Psychiatry Group based at the Max Planck UCL Centre for Computational Psychiatry and Ageing Research.^34^ The app can be used in any hand-held electronic device that runs on an Android or iOS operating system. The Brain Explorer app was created as a set of games set in space, designed to explore cognitive mechanisms, focusing on decision making and learning. In the games, participants are space explorers who have a mission to perform outer-planet activities. Participants were asked to complete tasks over the course of 5 different games (each set in a different planet) designed to collect data on specific domains: Treasure Hunt (decision making and information gathering), Milky Way (reward learning), Pirate Market (punishment learning), Space Observer (perceptual metacognition), and Scavenger (risk-taking/gambling). These games were selected to align with the functional magnetic resonance imaging (fMRI) protocol and assessments.

Using computational models to capture behavior from these games, the app aims to trace cognitive functioning and to determine how users differ in the way they act. During W3, participants were invited to download the Brain Explorer app and to finish all of the games during the 14-day period of remote data collection. Reminders were sent every other day to participants who did not finish the games after the first 7 days of the collection period.

#### Passive Sensing Data

In the present protocol, we used the Electronic Behavioral Monitoring (EBM) App (version 2.0), a custom-built application that has been previously used for passive sensing of adolescent mothers with depression in Nepal.^35^^,^^36^ The EBM App uses smartphone passive sensors to capture mobility patterns (both spatial, via GPS, and physical, via accelerometer/gyroscope) and ambient audio recordings (via microphone). The app was designed to capture these 3 types of smartphone data at 15-minute intervals from 6 am to 11:59 pm. Participants were instructed to download the EBM application and to log in with their phone numbers. The EBM also includes a timer that allows participants to interrupt data collection for a specified amount of time. In addition, participants were informed that no data are collected when the phone is turned off. Minimum requirements for installation are Android version 5.0 or above, a working microphone, and GPS; also, the phone must be capable of receiving a text message confirmation for logging into the application. During W2, participants who did not have a phone meeting the minimum requirements for EBM installation were offered a compatible smartphone for the 14-day period. To standardize the passive data collection using participants’ own smartphones, participants without a smartphone meeting the minimum requirements were excluded from EBM data collection at W3. Participants were asked to turn on the Internet connection and GPS on their phones for the 14 days of the study, as well as to enable the application to run in the background.

To complement the information from the passive movement and location data collected from the EBM app, data from GPS, accelerometer, and gyroscope features were also collected through the Google Location History (GLH) and Google Fit app at W3. GLH is a Google Account setting that accesses the smartphone’s GPS and saves the locations where the user has visited, capturing the user’s location based on the smartphone’s location services passively integrated into the Android operating system or with any other operating systems that have a Google application installed, such as Google Maps app. The Google Fit app is an open platform developed by Google Inc., which uses the smartphone sensors, such as accelerometer, gyroscope, and GPS system to detect changes in position (eg, moving from sitting to standing), various types of movement (walking, cycling, and others), several kinds of data (number of steps, walked distance, heart rate, and others), and different bouts of activity (time of each bout).^37^

Previous studies have used GLH services and Google Fit app to understand aspects of the health of individuals.^38^, ^39^, ^40^ In the present protocol, participants who have a Google account must have their GLH and Google Fit activated in order to generate data. Those who did not have a Google account or who did not have GLH and/or activity and movement history activated were invited to create an account and/or activate GLH (Google Maps) and Google Fit. GLH and Google Fit data were collected during the same period of EBM data collection in W3. In all cases, participants shared their data through Google Takeout, a free tool developed by Google Inc. to export Google data for backup. The DNs instructed the participants to install the Google apps, activate GLH, and share their data through Google Takeout. To make comparisons between the waves, participants who already had a Google account and had already activated GLH and Google Fit were also asked to share their retrospective data for the equivalent 14-day period following the collection of W0, W1, and W2.

#### Sleep and Circadian Rhythms: the CHRONO-IDEA Study

The bidirectional relationship between biological rhythms and depressive symptoms has been investigated,^41^, ^42^, ^43^ but is still far from being fully explored. The tools and techniques provided by chronobiology produce essential information to unveil the etiology, diagnosis, and prognosis of mental disorders.^44^, ^45^, ^46^ In clinical settings, several studies have observed consistent associations between greater depressive symptoms and lower activity rhythms,^47^ exposure to artificial light at night (ALAN),^48^ and sleep disturbances.^49^ Therefore, to investigate these features, the CHRONO-IDEA, an add-on study to IDEA-RiSCo, was designed to investigate questions related to chronobiology in the context of risk and presence of depression in adolescence. To this end, we collected data based on self-reports of sleep–wake cycle (ie, schedules, quality, and disturbances) and circadian phenotype (ie, chronotype), but also collected actimetry data. Actimetry is a passive method to assess rest–activity profiles using a wrist accelerometer. During W0, participants were invited to collect actimetry data for 14 consecutive days, right after the clinical assessment. At W3, actimetry data were collected for 23 consecutive days, starting concurrently with the remote collection period of the other digital phenotyping applications. In the presence of a researcher, the adolescent completed the instruments and was given the actimeter. At both waves, instruments were used to collect data on sleep–wake behavior on work(school) days and work(school)-free days, schedule preferences, sleep hygiene behaviors, and sleep difficulty, respectively (Table 1). Actimeters from ActTrust Condor are equipped with a luximeter and a thermometer, enabling the assessment of environmental light exposure, and peripheral body temperature, which were used in this study.

#### Other Measures of Interest

All individuals who participated in the W3 data collection at the Hospital de Clínicas de Porto Alegre Clinical Research Center also took part in the same collection procedure of peripheral biological samples performed at the baseline assessment. For participants who completed the clinical interview online at HCPA, anthropometric measures were collected immediately following the interview. For those who completed the clinical interview from home, anthropometric measures were collected on the same day as the neurobiological sample collection. Height, weight, waist circumference, hip circumference, and axillary temperature were collected following the same methodology reported in Kieling *et al.* (2021).^14^

To measure a range of pro- and anti-inflammatory cytokines and other immune-related markers, serum from whole blood (4 mL of blood using Vacutainer tube without anticoagulants) and plasma samples (4 mL of blood using a K3EDTA anticoagulant tube) were collected either in the morning or in the afternoon, depending on the time of clinical assessment. To perform gene expression analyses, RNA samples were also collected (5 mL of blood using two 2.5mL PAXGene tubes, PreAnalitix, Qiagen/BD Company). Detailed immune phenotyping analytic approaches are described in the IDEA-FLAME protocol.^50^

Participants took part in MRI data acquisition on the same day as the clinical assessment, using the same protocol as in W0, including structural and functional (gambling task, face-matching task, and resting-state) imaging acquisition.^14^^,^^51^, ^52^, ^53^ Throughout the study (baseline and follow-up waves), images were acquired from the same 3T Ingenia scanner (Koninklijke Phillips N.V., Netherlands), software version 5.3.1, and 16-channel head coil.

For W3, adolescents were also invited to provide hair samples for assessment of cortisol concentrations. Two hair strands were cut from the posterior vertex position of the head as close to the scalp as possible.^54^

### Data Management

All clinical data were collected and managed using the Research Electronic Data Capture (REDCap) platform hosted at HCPA.^55^^,^^56^ All self-report instruments were adapted to an electronic survey in REDCap; clinician-administered instruments (eg, K-SADS-PL) were also adapted, and their algorithms were fully implemented in REDCap.

The resulting database from the 14-day period of remote data collection during W2 and W3 underwent a data cleaning process to detect incomplete data or other inconsistencies. All information collected by the IDEABot was uploaded using the encrypted WhatsApp server to our own secure server. Afterward, all audio files were transcribed and subsequently stored in the cloud, encrypted and without identifying information.^31^ Brain Explorer data were stored on servers at University College London (UCL).

The GPS and accelerometer/gyroscope data from the EBM app were first stored locally on the participants’ smartphones as a CSV file, whereas audio files were stored in m4a format. All participant data could be accessed (and deleted) from the phone. In the presence of an Internet connection, the application automatically uploaded participant data to our private server. The passive data from the EBM app, GLH, and Google Fit were stored on a private, secure online drive using Secure Socket Layer (SSL) encrypted connection, ensuring total confidentiality, integrity, and accuracy of the data being transmitted. The processing and analysis of passive sensing data will be published in future publications.

### Cohort Retention Rates

Recruitment for the last follow-up wave ended in September 2022. Data collection, cleaning, and preparation are complete, and data analysis is currently underway. Baseline characteristics of the IDEA-RiSCo sample have been presented elsewhere.^14^ In each of the 3 subsequent waves, participants were considered as included if at least 1 measure of depressive symptoms (from either a participant or caregiver questionnaire) was completed.

Over the 3-year follow-up, the overall retention rates at W1, W2, and W3 were 96% (n = 144), 94% (n = 141), and 88% (n = 132). Of the participants, there were 83.3% (n = 125) who completed at least 1 data point for each of the 4 data collection timepoints. Although loss to follow-up was higher in W3, rates were not significantly different between sexes and risk groups for all 3 waves in the follow-up (*p* > .05). Refusals were the main source of loss for all 3 waves, and only 2 participants at each wave were not found during the contact period.

A majority of the included participants, 80.8% (n = 114) at W2 and 97% (n = 128) at W3, took part in some aspect of the 14-day period of digital data collection. Of these, 70.2% and 89.1% used their own smartphone for all digital measures during W2 and W3, respectively. Preliminary results of the IDEABot have been published in a specific paper.^31^

Among the full sample, 86% (n = 129) completed the clinical assessment and underwent K-SADS-PL and other clinician-administered measures at the endpoint (W3). Of these, 93.8% (n = 121) had biological material collected (blood/hair samples), and 76.7% (n = 98) underwent MRI acquisition. Retention rates were not significantly different between study groups for both neurobiological collection data (*p* > .05). The main reason for the missing MRI data was the presence of fixed metal accessories (mostly dental braces), a contraindication to the procedure, for several participants (n = 20) at W3.

### Participants’ Perspectives

Eight of the 10 adolescents included at the end of the baseline assessment period who met criteria for a diagnosis of depression were invited to participate in semi-structured interviews to explore their experience as research participants.^14^^,^^57^ In their accounts, adolescents reported limited understanding of the purposes of the research but stressed that the overall study process was positive; in fact, some considered that participating was even beneficial. Among the motivators for study participation, adolescents reported seeing the research as a way of being helped and of having feelings and difficulties acknowledged. Also, helping other adolescents who may be struggling with depression was mentioned as a great motivator for participating in the research.^14^

In the third wave of follow-up, we were also interested in how participants perceived the data collection process, especially the new technologies added to the research protocol. Thus, we included in the IDEABot a question regarding the participants’ experiences with the tool and the completion of the research process.^31^

Overall, participants endorsed the familiarity with the research process and team (given the length of data collection over 3 years) as a motivator for sustained engagement and overall appraisal of the process:

“For me, it was very normal [to complete data collection] because I had already participated in the research, so I knew how it worked.” (Girl, 19 years)

“It’s always a pleasure to participate in all the surveys we do, in everything, all the stages, whether face-to-face or online.” (Girl, 19 years)

The idea of helping research and other adolescents was also mentioned. At baseline, this was considered a strong motivator for study engagement. In the follow-up, adolescents also endorsed this motive; however, they also expressed doubt, revealing an overall limited understanding of the goal of data collection:

“I know that I will be helping some people, I mean, maybe not, but I think I will.” (Boy, 20 years)

The study design also seems to have contributed to encouraging participants to share private aspects of their lives with the researchers. Consistent with the baseline round, participants endorsed the positive aspects of data collection as providing a space for reflection and regulation regarding their own emotions, even if the IDEABot represented a different medium for doing so.

“It was a cool, innovative experience, because I don’t like showing my feelings so much, talking about things to people a lot and it ended up that I had to do this here, kind of leaving my comfort zone, you know. So I think that that was cool; it was, it made me evolve and I think I'll use that as an example. Not just talking here with the bot, but talking about something with, with people, expressing my feelings. I think this here served as a lesson.” (Boy, 19 years)

Conversely, some expressed annoyance at the mode of data collection—both in and outside clinical settings, using not only questionnaires, but also audio recordings:

“Ah, it was a bit annoying having to keep talking, talking, talking, talking. Sometimes I had to give details about everything, even when I had nothing else to say, so I had to keep going on with useless and unnecessary things like I'm doing now. It was basically boring, boring, boring, boring, answering that.” (Boy, 20 years)

## Discussion

This protocol paper describes the methods and procedures for 3 follow-up waves of a prospective study to assess the longitudinal course of depressive symptomatology among 150 adolescents stratified for risk of developing depression and presence of depression, and presents the initial results regarding feasibility of the procedures and retention. Future studies will thus focus on analyzing the wealth of data successfully captured through the application of this protocol.

The IDEA-RiSCo study is an innovative project involving deep phenotype analysis using technology-mediated data collection combined with traditional clinical assessments and neurobiological approaches. Despite the complexity of collecting longitudinal data among adolescents, the prospective follow-up achieved a low attrition rate (12%) at endpoint (W3). In contrast, similar studies with adolescents and young adults at high risk for psychosis have reported attrition rates of about 30%.^58^ Therefore, the present protocol might offer insights about the feasibility of longitudinal prospective data collection in a phenotypically refined sample of adolescents.

Several studies have relied on sensing data collected from smartphones to predict mental health and well-being in adults.^59^, ^60^, ^61^ It has been theorized that low-level sensor data may correlate with depression severity in young adults,^62^ and behavioral data have been shown to predict symptoms of depression and post-traumatic stress disorder.^63^ Little research, however, has focused on adolescent populations, even though smartphone use is widespread in this age group.^64^ To address this gap, the present protocol included mobile-mediated active and passive sensing data collection at 2 of the follow-up waves. We also proposed a frugal innovation tool (IDEABot) to collect repeated measures of depressive symptomatology and brief spontaneous audio recordings.^31^ The IDEABot uses WhatsApp as the interface for data collection, addressing the respondent burden, which may contribute to self-selection bias and selective nonresponse.^65^ With this study, we hope to contribute to a common understanding of the applicability of data collection using smartphones in the adolescent population. This will provide interesting insights on how we can better collect, manage, and analyze robust data from smartphones to sample from adolescents’ experiences.

Furthermore, the integration of digital and traditional methods presents a promising frontier in mental health, particularly given its underexplored potential within adolescent populations. Technological innovations in active and passive sensing devices have the potential to revolutionize people-centric sensing, largely due to the transformation of the near-ubiquitous mobile phone into a dynamic sensing device.^66^ This evolution, coupled with the computational prowess and pervasive presence of smartphones, facilitates the collection of digital phenotypes that might echo individuals’ lived experiences within their natural environments.^67^ Consequently, digital phenotyping using smartphones and other digital tools can notably augment the precision of depression diagnosis.^68^ Moreover, despite the challenges of a potential digital divide,^69^ the use of smartphones for data collection may represent a scalable, cost-effective data gathering strategy. This potential enhancement could advance early identification of mental health conditions, as well as refine treatments by providing parameters for personalized interventions.^70^

A number of studies have been published based on IDEA-RiSCo baseline data. Findings from neuroimaging studies have demonstrated associations of reward- and threat-related neural function and frontolimbic network topology with depression and with high-risk status for developing depression.^51^, ^52^, ^53^ Chronobiological findings include strategies to deal with missing data in actimetry,^71^ and the relationship among different risk groups and sleep variables, motor activity rhythms, and exposure to artificial light at night.^72^ Furthermore, a qualitative study was also published to explore the adolescents’ initial reactions after receiving a clinical diagnosis of MDD.^57^ Additional publications are currently underway, but the limitations inherent to the cross-sectional focus of the studies using only baseline data must be recognized.

Nevertheless, by setting a solid foundation for subsequent detailed analyses of the course of depressive symptomatology and neurobiological features across risk groups, the present protocol represents a significant advancement for the understanding of adolescent depression. The methodological framework that we established has enabled consistent data collection across 4 time points and opens several avenues for research. First, the predictive validity of the IDEA-RS will be assessed evaluating, in each group, the proportion of participants developing depression 3 years post-baseline. Also, it will be possible to examine whether neural function patterns, sleep, and biological rhythm variations in the LR and HR groups augment depression development prediction. In a broader context, it is essential to understand the potential for nuanced analyses in early- vs later-onset MDD. By identifying intraindividual changes in neurobiological and clinical attributes before and after the onset of depression, researchers can better pinpoint risk factors and intervention targets. Moreover, future studies should analyze whether there are any differences in earlier-onset vs later-onset MDD, identifying intraindividual changes in terms of neurobiological and clinical characteristics before and after developing depression.

Thus, this study’s strengths lie not only in its potential to advance our understanding of risk profiles, symptomatic progression, and neurobiological patterns over time in Brazilian adolescents, but also in its methodological approach. The blend of active and passive sensing data collection paves the way for nested timeframe analyses. While we use a 3-year follow-up within a cohort framework, we simultaneously delve into intensive longitudinal datasets, with 28 days of repeated measures across 2 years. Such a comprehensive approach aids in investigating intraindividual changes and variability across time.^73^

This dual approach, which allows for long-term intraindividual changes to be juxtaposed with short-term depressive symptomatology data, represents a holistic view. It not only aids in individual-focused analyses but also provides insights into group dynamics, highlighting how group and individual factors interplay.^74^ Furthermore, pairing innovative collection methods with neurobiological measures will undoubtedly propel our comprehension of the neural and immune intricacies underlying adolescent depression. This strategy offers an exciting avenue for future research, facilitating data triangulation and allowing for method-wise comparison of neurobiological data. We believe that the steps that we have taken in this study will act as a cornerstone for subsequent research, elucidating the intricate web of factors contributing to adolescent depression.

However, this study is not without limitations, which are addressed below. The sample size may limit the power to compare some uncommon outcomes or parameters with higher variability between risk groups. However, we believe that we have mitigated this limitation by recruiting homogeneous groups, recognizing the heterogeneity of the group without depression, applying repeated measures, and ensuring a high level of detail in the information collected—which included in-depth clinical assessments—for each individual.

Technological limitations include that the digital data collection may be affected by limitations such as malfunction of apps, adolescents forgetting to charge their phone, or devices not retaining charge.^75^^,^^76^ Moreover, concerns about privacy, interference with daily activities, and cultural issues may also influence the participants’ attitudes toward the use of technology.^76^ Our study has attempted to mitigate gaps in digital data collection by daily monitoring of the active and passive data acquisition with notifications to the participants if abnormalities were detected. We also included a collaborative approach in the development and implementation of all technology-mediated data collection procedures.^31^

Nonadherence is another potential shortcoming. Experimental fatigue associated with completing repeated measures might result in missing data during data collection across the waves.^77^ Furthermore, longitudinal studies may also have unintended effects, such as participants engaging in healthier behaviors when tracking their own emotions and health.^78^ During the 14-day remote data collection period, we conducted naturalistic data collection processes to reduce the burden on participants. We also attempted to reduce participant burden by keeping daily assessments brief and providing adaptations in the collection settings (eg, assessments from home for those who were not able to come to hospital).

The COVID-19 pandemic represents another potential limitation of this study. The coronavirus pandemic emerged during the follow-up period of this protocol. Recently, some studies have pointed out that the pandemic has influenced the mental health of young individuals.^79^, ^80^, ^81^ To study the impact of the pandemic, we incorporated an adapted version of the The CoRonavIruS Health Impact Survey (CRISIS) during W3.^82^ In addition, all data collection procedures during W1 and W2 and part of those conducted in W3 were performed online. In this context, the adequate retention rates achieved even in the context of the pandemic could be considered a strength of this study.

In regard to the adolescents’ experience of participating in the follow-ups, some important aspects stand out. First, despite the familiarity with the research process, participants still expressed limited understanding of the research goals and objectives. This finding raises the issue of the complexification of the protocol and inclusion of innovative approaches as an additional concern regarding how to translate research into meaningful information for adolescents. Second, an interesting observation about the motivation for participating in the study was the notion that, as research participants, the adolescents would be able to help research and other adolescents. Data suggest altruism as a strong motivator for sustained engagement in research protocols, which is consistent with previous research that can be leveraged for future research.^83^ In addition, meaningful rapport with the research team was an important aspect for engagement at both baseline and follow-up. Finally, participants also endorsed the use of the research process as being important for validating and reflecting upon their emotional states, which was described as therapeutic in its own right. However, we have few data on how this effect was experienced in depth by the adolescents, and thus future research is required to explore this further.

Despite the global importance of depression in adolescence, there are significant gaps in the quality and number of studies addressing the course of the disorder in this age group.^13^ This is particularly meaningful in LMICs, where the majority of the world’s adolescent population lives and the evidence on mental health in youth is scarce.^14^ In this regard, the present protocol has shown the feasibility of conducting a prospective follow-up study with a risk-enriched cohort of adolescents in a middle-income country. Moreover, this study has contributed to knowledge regarding the integration of mobile technology with traditional methodologies to improve intensive longitudinal data collection. Furthermore, we hope to support additional studies that in the long term could increase the understanding of the clinical and neurobiological trajectories of the complex phenomenon of depression in adolescence.
