# Supplementary material for: Prospective Follow-Up of Adolescents With and at Risk for Depression: Protocol and Methods of the Identifying Depression Early in Adolescence Risk Stratified Cohort Longitudinal Assessments
Source: JAACAP Open. 2023 Dec 14;2(2):145–59. doi: 10.1016/j.jaacop.2023.11.002 (PMC11163476; doi:10.1016/j.jaacop.2023.11.002)
Supplement: Supplements 1, 2, and 3 [file mmc1.docx]

**Supplemental Materials**

[**Supplement 1** Animated video explaining the functioning of the EBM App](https://drive.google.com/file/d/1heG0Bf_3msb0jRL-45I-_N2kFME2EHfG/view?usp=share_link)

[**Supplement 2** Animated video explaining the functioning of the IDEABot](https://drive.google.com/file/d/1bM6fGakGb9yDyt9rHktukCs5T5m6lELs/view?usp=share_link)

[**Supplement 3** Animated video explaining the functioning of the BrainExplorer App](https://drive.google.com/file/d/10qeFaqSCqLcfBrr180ifqbYcHj9aJ1X7/view?usp=share_link)
